# Refractory postoperative *Staphylococcus hominis* bacteremia in a patient with an ACTH-producing pancreatic neuroendocrine neoplasm: a case report

**DOI:** 10.1186/s40792-022-01485-8

**Published:** 2022-06-29

**Authors:** Ryuta Muraki, Yoshifumi Morita, Kyota Tatsuta, Shinya Ida, Ryo Kitajima, Amane Hirotsu, Makoto Takeda, Hirotoshi Kikuchi, Yoshihiro Hiramatsu, Atsuko Fukazawa, Go Kuroda, Keisuke Kakizawa, Hiroya Takeuchi

**Affiliations:** 1 Department of Surgery, Hamamatsu University School of Medicine, 1-20-1 Handayama, Higashi-ku, Hamamatsu, Shizuoka 431-3192 Japan; 2 Department of Perioperative Functioning Care & Support, Hamamatsu University School of Medicine, 1-20-1 Handayama, Higashi-ku, Hamamatsu, Shizuoka 431-3192 Japan; 3 Department of Gastroenterological Surgery, Iwata City Hospital, 512-3 Ohkubo, Iwata, Shizuoka 438-8550 Japan; 4 Department of Internal Medicine, Hamamatsu University School of Medicine, 1-20-1 Handayama, Higashi-ku, Hamamatsu, Shizuoka 431-3192 Japan

**Keywords:** *Staphylococcus hominis*, ACTH-producing pancreatic neuroendocrine neoplasm, Catheter-associated bacteremia

## Abstract

**Background:**

*Staphylococcus hominis* (*S. hominis*) is an opportunistic pathogen that is often highly resistant to antibiotics and is difficult to treat. In patients diagnosed with an adrenocorticotropic hormone (ACTH)-producing tumor that compromises the immune system due to hypercortisolemia, cancer treatment and infection control should be considered simultaneously. This report presents a case of refractory postoperative *S. hominis* bacteremia requiring the prolonged administration of several antibiotics in a patient with an ACTH-producing pancreatic neuroendocrine neoplasm (pNEN).

**Case presentation:**

A 35-year-old man visited a neighboring hospital for a thorough examination after experiencing weight gain and lower limb weakness for several months. Enhanced computed tomography revealed a pancreatic tail tumor and bilateral adrenal enlargement. Elevated plasma ACTH and serum cortisol were noted. Biopsy under endoscopic ultrasonography revealed the tumor as an ACTH-producing pNEN. The patient was transferred to our hospital for further treatment. Pneumocystis pneumonia was noted and treated with sulfamethoxazole and adjunctive glucocorticoids. Hypercortisolism was controlled with metyrapone and trilostane. Somatostatin receptor scintigraphy and ethoxybenzyl magnetic resonance imaging detected other lesions in the pancreatic head. A total pancreatectomy was performed given that the lesions were found in both the pancreatic head and tail. Plasma ACTH and serum cortisol levels decreased immediately after the resection. Pathological examination revealed that the pancreatic tail tumor was NEN G2 and T3N1aM0 Stage IIB and the pancreatic head lesions were SSTR-positive hyperplasia of the islet of Langerhans cells. On postoperative day 11, catheter-associated bacteremia occurred. Initially, meropenem hydrate and vancomycin hydrochloride were administered empirically. *S. hominis* was identified and appeared sensitive to these antibiotics according to susceptibility testing. However, *S. hominis* was repeatedly positive in blood cultures for more than one month, despite treatment with several antibiotics. Eventually, with the combined use of three antibiotics (meropenem hydrate, vancomycin hydrochloride, and clindamycin phosphate) for more than 3 weeks, the *S. hominis*-associated bacteremia improved. He was discharged 79 days after surgery.

**Conclusions:**

Our patient with an ACTH-producing pNEN was immunocompromised and needed meticulous attention for infectious complications even after successful tumor removal. Specifically, *S. hominis* bacteremia in such patients demands intensive treatments, such as with combinational antibiotics.

## Background

*Staphylococcus hominis* (*S. hominis*) is one of the most abundant microbial species on the human skin; it is an opportunistic pathogen that is found in the blood of neonates and patients on immunosuppressants [1, 2]. An adrenocorticotropic hormone (ACTH)-producing pancreatic neuroendocrine neoplasm (pNEN) and the resultant hypercortisolism carry a poor prognosis, with a short median survival and a high incidence of infectious complications [3, 4]. We present the case of a patient with an ACTH-producing pNEN who developed a postoperative *S. hominis* infection that was refractory to even sensitive antibiotics.

## Case presentation

**Fig. 1:**
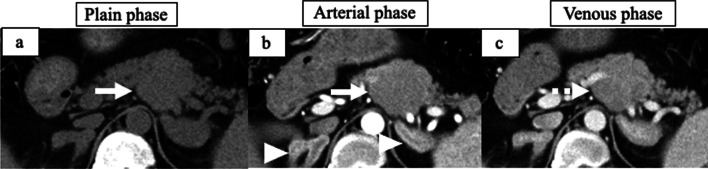
Enhanced CT imaging. **a** Plain CT reveals the tumor located in the pancreatic tail (55 mm in diameter; white arrow). **b** Enhanced CT reveals the tumor with enhancement (white arrow) and bilateral adrenal enlargement (white arrowhead). **c** Enhanced CT reveals the tumor persisting with weak enhancement (dotted white arrow). *CT* computed tomography

**Fig. 2:**
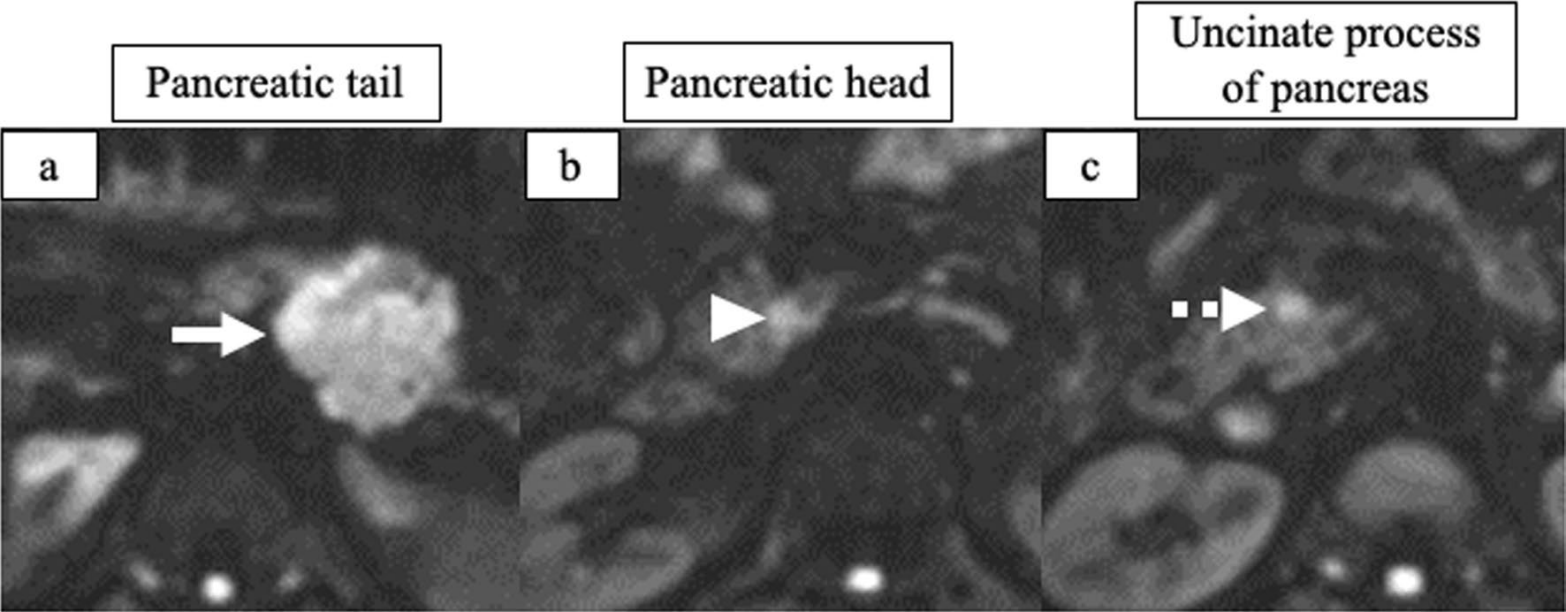
MRI diffusion-weighted imaging. **a** The tumor located in the pancreatic tail is seen (white arrow). **b** The tumor located in the pancreatic head is seen (white arrowhead). **c** The tumor located in the uncinate process of the pancreas is seen (dotted white arrow). *MRI* magnetic resonance imaging

**Fig. 3:**
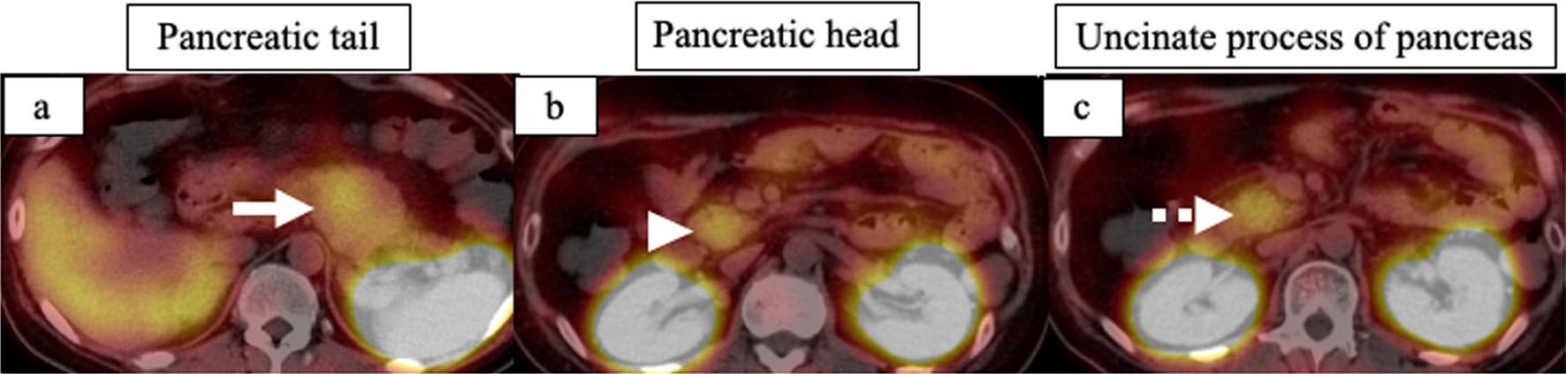
SRS. **a** SRS reveals a high accumulation of radioactive tracer in the pancreatic tail (white arrow). **b** SRS reveals a high accumulation of radioactive tracer in the pancreatic head (white arrowhead). **c** SRS reveals a high accumulation of radioactive tracer in the uncinate process of the pancreas (dotted white arrow). *SRS* somatostatin receptor scintigraphy

**Fig. 4:**
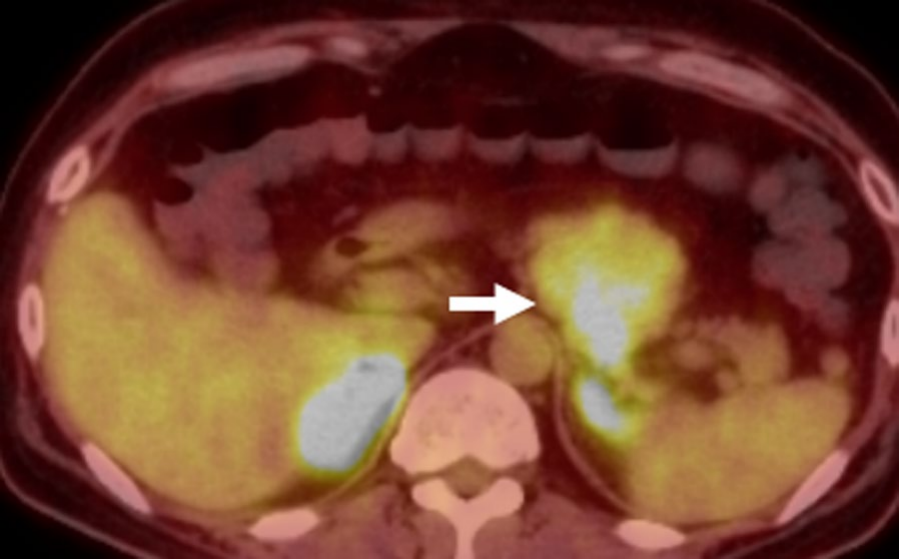
FDG PET–CT imaging. FDG PET–CT reveals FDG deposition in the pancreatic tail: the maximum standardized uptake value is 16 (white arrow). FDG PET–CT: 2-[18F] fluoro-2-deoxy-d-glucose positron emission tomography computed tomography

**Fig. 5:**
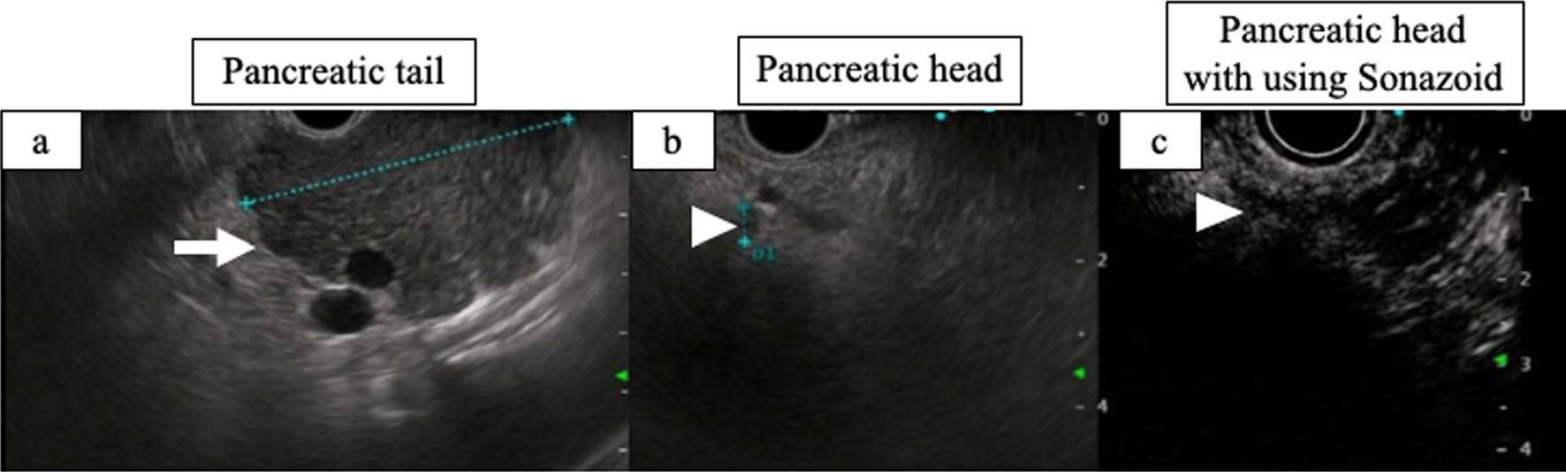
Endoscopic ultrasonography. **a** A hypoechoic lesion, 55 mm in diameter, is detected in the pancreatic tail (white arrow). **b** A hypoechoic lesion, 5 mm in diameter, is detected in the pancreatic head (white arrowhead). **c** A hypoechoic lesion is not enhanced with Sonazoid (white arrowhead)

**Fig. 6:**
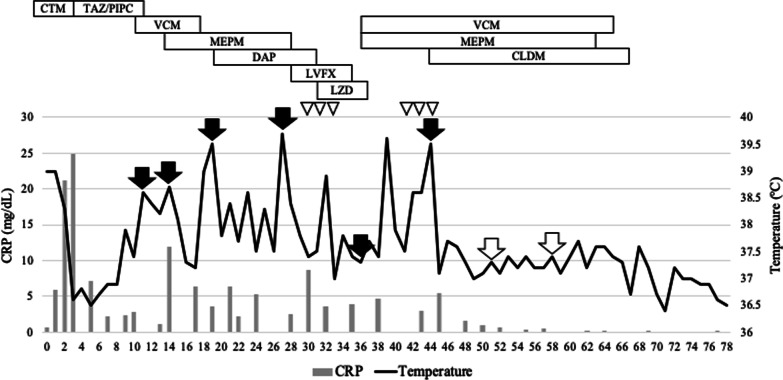
The postoperative course and antibiotic treatments. The black arrow indicates *S. hominis*-positive blood culture, while the white arrow indicates *S. hominis*-negative blood culture. The white arrowhead indicates the timing of intravenous immunoglobulin administration. *CTM* cefotiam hydrochloride, *TAZ/PIPC* tazobactam, piperacillin hydrate, *VCM* vancomycin hydrochloride, *MEPM* meropenem hydrate, *DAP* daptomycin, *LVFX* levofloxacin hydrate, *LZD* linezolid, *CLDM* clindamycin phosphate, *CRP* C-reactive protein

**Fig. 7:**
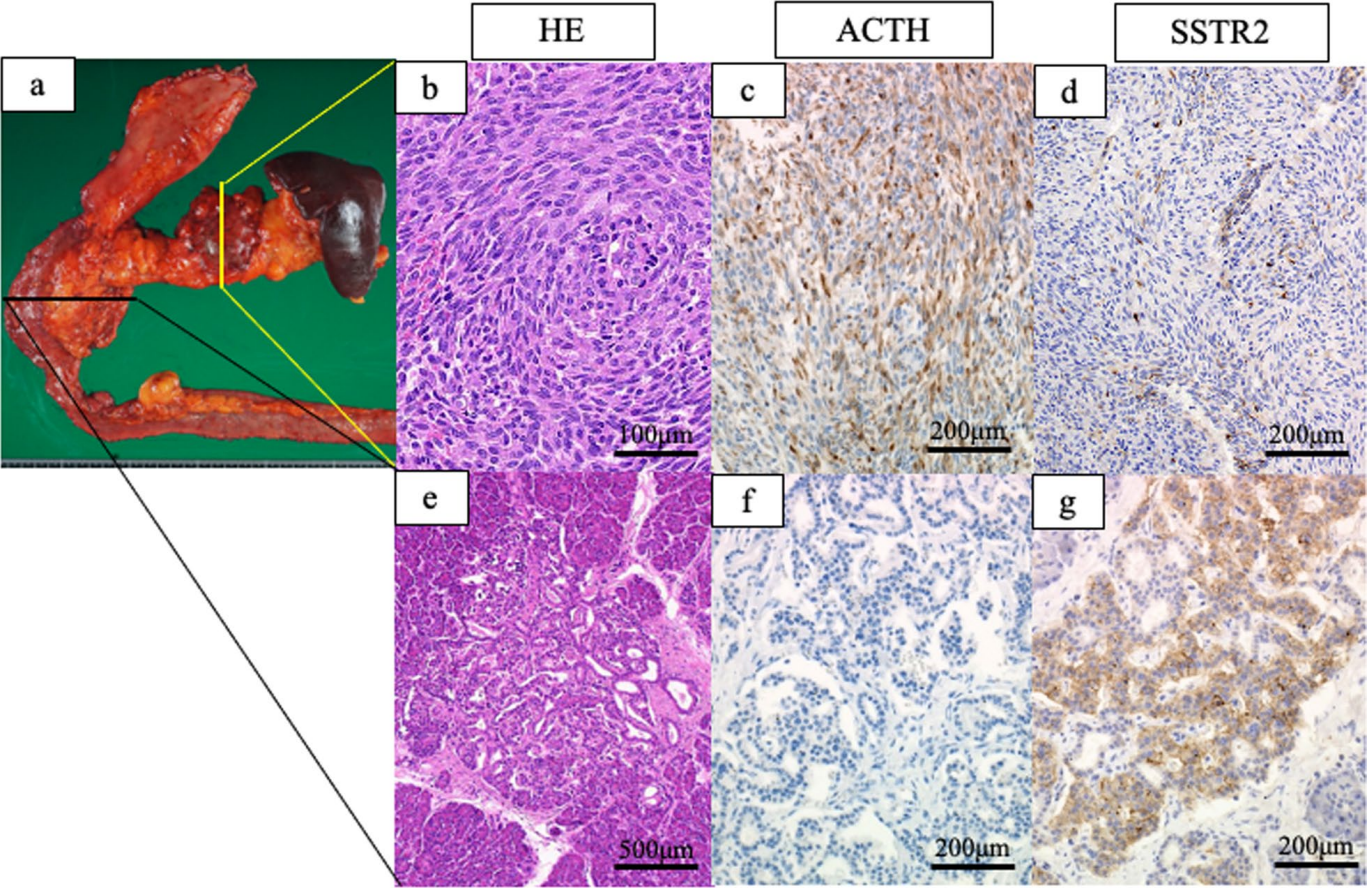
Histopathological examination. **a** The tumor located in the pancreatic tail is 6 cm in diameter. **b** The tumor located in the pancreatic tail contains rosette-like components when viewed microscopically. **c** Immunohistochemical examination shows ACTH positivity. **d** SSTR2 expression in the tumor is weakly positive. **e** Multiple lesions of islet of Langerhans cell hyperplasia are seen in the head of the pancreas. The maximal size of the islets is 2 mm. **f** Immunoreactivity for ACTH is negative. **g** The tumor cells are positive for SSTR2. *HE* hematoxylin and eosin stain, *ACTH* adrenocorticotropic hormone, *SSTR* somatostatin receptor

A 35-year-old man with hypertension experienced weight gain and lower limb weakness for several months. He first visited a neighboring hospital for a thorough examination, where he presented with a Cushingoid appearance that included a moon-face, proximal limb muscles weakness, central obesity, and thinning of the skin. He had no family history associated with multiple endocrine neoplasia type 1. Enhanced computed tomography (CT) revealed a pancreatic tail tumor (55 mm in diameter) and bilateral adrenal enlargement (Fig. 1). Elevated plasma ACTH at 791 pg/mL (normal range: 7.2–63.3 pg/mL) and serum cortisol at 121 µg/dL (normal range: 3.7–19.4 µg/dL) were also noted. The patient’s cortisol level was not suppressed by the dexamethasone suppression test, and his ACTH level was not suppressed by either the dexamethasone suppression test or the octreotide test. Biopsy under endoscopic ultrasonography revealed the tumor to be an ACTH-producing pNEN. The patient was then referred to our hospital for further treatment. The patient had pneumocystis pneumonia and was treated with sulfamethoxazole and an adjunctive glucocorticoid (60 mg of prednisone per day), which were gradually tapered and discontinued. Concurrently, his hypercortisolism was controlled with metyrapone and trilostane. In addition to the pancreatic tail tumor, ethoxybenzyl magnetic resonance imaging (MRI) and somatostatin receptor scintigraphy detected two new lesions in the pancreatic head (Figs. 2, 3). However, 2-[18F] fluoro-2-deoxy-d-glucose (FDG) positron emission tomography (PET)–CT revealed FDG deposition in the pancreatic tail tumor alone (Fig. 4). Contrast-enhanced endoscopic ultrasonography revealed a lesion (55 mm in diameter) in the pancreatic tail and another lesion (5 mm in diameter) without enhancement in the pancreatic head (Fig. 5). Taking the possibility of intrapancreatic metastasis or multicentric tumorigenesis into consideration, a total pancreatectomy was planned, pending confirmation of the pancreatic head lesion by an intraoperative ultrasound (IOUS) examination. The IOUS examination confirmed the lesion in the pancreatic head, and a total pancreatectomy combined with splenectomy was performed. The operation time was 592 min and the intraoperative blood loss was 1,327 mL. Central obesity and fragile adipose tissue made surgery difficult. The plasma ACTH and serum cortisol levels normalized the day after the operation (8.5 pg/mL and 6.7 µg/dL, respectively). Cefotiam hydrochloride was administered for 2 days as a prophylactic antibiotic. A high dose of hydrocortisone (225 mg/day) was administered during the perioperative period to prevent an adrenal crisis; it was gradually tapered to physiologic replacement doses. For blood glucose control after the total pancreatectomy, the patient was administered a continuous venous infusion of insulin during fasting and multiple daily insulin injections after the start of meals. Consequently, the blood glucose level was maintained at approximately 200 mg/dL. Because the C-reactive protein level was elevated (21.25 mg/L) on postoperative day (POD) 2, tazobactam and piperacillin hydrate was administered as empirical therapy. However, central venous catheter-associated bacteremia occurred on POD 11; therefore, the patient was secondarily administered with meropenem hydrate and vancomycin hydrochloride (Fig. 6). The central venous catheter inserted in the operating room had been removed after the first blood culture was collected. *S. hominis* was identified in both catheter and blood cultures, and susceptibility testing revealed that the organism was sensitive to the already administered antibiotics (Table 1). However, despite the administration of several antibiotics that the bacterium is susceptible to (such as daptomycin, levofloxacin hydrate, or linezolid), *S. hominis* was repeatedly positive in the blood cultures for more than a month. Intravenous immunoglobulin (5000 mg/day) was also administrated as adjunctive therapy for bacteremia during PODs 30–32 and 42–44. Specialized examinations, including enhanced CT, ultrasound cardiography, and FDG-PET–CT, were performed to investigate the source of the infection. Various bacterial cultures were performed using the bile, urine, and drainage fluid. However, except for the blood, no other apparent source was detected. Eventually, after more than 3 weeks of the combined administration of three antibiotics (meropenem hydrate, vancomycin hydrochloride, and clindamycin phosphate), the *S. hominis*-associated bacteremia improved. The patient was discharged 79 days after the surgery. Pathological examination revealed that the pancreatic tail tumor was NEN G2, T3N1aM0 Stage IIB with ACTH (+), somatostatin receptor (SSTR2) (+), and a Ki-67 of 15%. The pancreatic head lesion was identified as SSTR-positive hyperplasia of the islet of Langerhans cells (Fig. 7). The patient did not receive any postoperative adjuvant therapy. Eight months after the surgery, scheduled enhanced CT revealed a recurrence in the para-aortic lymph nodes; furthermore, the plasma ACTH level, which had decreased to within its normal range after the pancreatectomy, was elevated to 153 pg/mL. The lymph node recurrence was resected, because these lesions were resectable. Pathological examination indicated that the para-aortic lymph node tumors were NEN G3 with ACTH (−), SSTR2 (+), and a Ki-67 of 20%. Soon after the second operation, multiple recurrences in the lymph nodes around the common hepatic artery, liver, and peritoneum occurred. After the para-aortic lymph node resection, the plasma ACTH level had dropped to 35.8 pg/mL; however, it increased again to 121 pg/mL at the time the multiple recurrences were detected. Currently, i.e., 14 months after his initial surgery, the patient is alive and is preparing to undergo chemotherapy with streptozocin and fluorouracil for the NEN G3.

**Table 1:** Results of the susceptibility testing of *Staphylococcus hominis*

Antibiotics	POD 11		POD 14		POD 19		POD 27		POD 35		POD 44	
	MIC	Interpretation	MIC	Interpretation	MIC	Interpretation	MIC	Interpretation	MIC	Interpretation	MIC	Interpretation
Cefazolin sodium	< 2	S	< 2	S	< 2	S	< 2	S	< 2	S	< 2	S
Ampicillin/sulbactam	< 8	S	< 8	S	< 8	S	< 8	S	< 8	S	< 8	S
Amoxicillin/clavulanate	< 2	S	< 2	S	< 2	S	< 2	S	< 2	S	< 2	S
Levofloxacin hydrate	< 0.5	S	< 0.5	S	< 0.5	S	< 0.5	S	< 0.5	S	< 0.5	S
Meropenem hydrate	< 1	S	< 1	S	< 1	S	< 1	S	< 1	S	< 1	S
Daptomycin	< 0.25	S	< 0.25	S	< 0.25	S	< 0.25	S	< 0.25	S	< 0.25	S
Linezolid	< 1	S	< 1	S	< 1	S	< 1	S	< 1	S	< 1	S
Vancomycin hydrochloride	< 0.5	S	< 0.5	S	< 0.5	S	< 0.5	S	1	S	< 0.5	S
Clindamycin phosphate	< 0.25	S	< 0.25	S	< 0.25	S	< 0.25	S	< 0.25	S	< 0.25	S

*POD* postoperative day, *MIC* minimum inhibitory concentration, *S* susceptible, *I* intermediate

## Discussion

We report the case of a patient with an ACTH-producing pNEN who suffered from postoperative infection with *S. hominis* even after complete resection of the tumor; our observations indicated that meticulous attention should be paid to the management of *S. hominis*-associated bacteremia. Herein, we have discussed why the patient remained immunocompromised even after tumor resection and how *S. hominis* bacteremia can be treated in cases similar to the present one.

With a 5-year survival rate of 35%, ACTH-producing pNENs have a high metastatic capacity and high postoperative mortality rates; compared with other functioning and nonfunctioning pNENs, ACTH-producing pNENs have a poorer prognosis [5, 6]. Although the optimal primary treatment is complete resection, most patients are diagnosed in locally advanced or metastatic stages [6]. In addition, surgical management was previously thought to be challenging, due to the patients’ frequently poor general conditions. In one previous case report, two patients with ACTH-producing pNENs without metastasis underwent surgery and died of surgery-related and hypercortisolism-related complications (one from pulmonary infection and one from abdominal infection) [6]. Another report described the case of a patient with an anatomically resectable ACTH-producing pNEN that could not be resected due to an uncontrolled infection; the patient died due to sepsis [7]. Based on these reports, hypercortisolism contributes to severe or opportunistic infections that lead to high morbidity and mortality rates [8]. In the present case, the preoperative medical treatment for controlling hypercortisolism and pulmonary infection was adequate; however, the patient’s postoperative course was protracted. Concomitant splenectomy may influence the patients’ immune capacity. Additionally, the patients may suffer from hypogammaglobulinemia, which is caused by endogenous or therapeutic hypercortisolism and leads to a decreased immunoglobulin G (IgG) production and increased IgG catabolism [9, 10]. The low level of IgG may persist even after discontinuation of steroid treatment [11]. This mechanism could potentially explain why the patient’s immune dysfunction was prolonged, although the IgG level was not measured in this case. An important aspect of ACTH-producing pNEN management is to treat the patients carefully, even after a successful tumor resection, because they are still in an immunocompromised state.

Evidence on the systemic treatment of recurrent ACTH-producing pNENs is lacking due to their rarity [12, 13]. We believe that the somatostatin analog administered to our patient was ineffective because his ACTH level was not suppressed by octreotide preoperatively. A previous study recommended adjuvant chemotherapy for locally advanced pNENs in cases with lymph node metastasis; however, unfortunately, that study did not include cases with ACTH-producing pNENs [14]. A report has suggested that an aggressive surgical management is reasonable for the primary and metastatic lesions of this disease, because it is optimal for controlling endocrinal abnormalities and prolonging survival [12, 13, 15]. In the present case, we performed an aggressive resection even for metastatic lesions and did not adopt adjuvant chemotherapy.

*Staphylococcus hominis*, a coagulase-negative staphylococcal strain, is a common organism that is involved in nosocomial bacteremia due to the increasing use of medical devices, such as intravenous catheters, vascular grafts, prosthetic heart valves, and devices used in the treatment of joint disease [16]. Previous reports indicate that almost all *S. hominis* isolates carry the *mecA* gene, which resides within a mobile genetic element called the staphylococcal cassette chromosome mec [1, 17]. As a result of the expression of this gene, *S. hominis* is highly resistant to methicillin. Moreover, *S. hominis* often produces biofilms, which worsen antibiotic resistance and require high doses of antibacterial agents [1]. According to one in vivo study, *S. hominis* exhibited a cytotoxic effect on human epithelial cells, thereby inducing the destruction of host tissues and facilitating bacterial invasion [17]. Considering this, the strategy against *S. hominis* sometimes requires the administration of multiple antibiotics, including clindamycin phosphate, over an extended period. In this case, there was no evidence of drug resistance to any of the administered antibiotics in the susceptibility testing. If multiple antibiotics, including clindamycin phosphate, had been administered consistently for an extended period, the *S. hominis* infection could have been resolved earlier.

## Conclusions

In this case, our patient with an ACTH-producing pNEN was immunocompromised and required meticulous monitoring for *S. hominis*-associated infectious complications even after a successful tumor removal. *S. hominis* bacteremia in such cases demands intensive treatments, such as combinational antibiotics.

## Data Availability

Not applicable.

## Abbreviations

*S. hominis*: *Staphylococcus hominis*
ACTH: Adrenocorticotropic hormone
pNEN: Pancreatic neuroendocrine neoplasm
CT: Computed tomography
MRI: Magnetic resonance imaging
FDG: 2-[18F] Fluoro-2-deoxy-d-glucose
PET: Positron emission tomography
IOUS: Intraoperative ultrasound
POD: Postoperative day
SSTR: Somatostatin receptor
